# Supplementary figures and images for: Tudor-domain containing protein 5-like promotes male sexual identity in the Drosophila germline and is repressed in females by Sex lethal
Source: PLoS Genet. 2019 Jul 22;15(7):e1007617. doi: 10.1371/journal.pgen.1007617 (PMC6645463; doi:10.1371/journal.pgen.1007617)

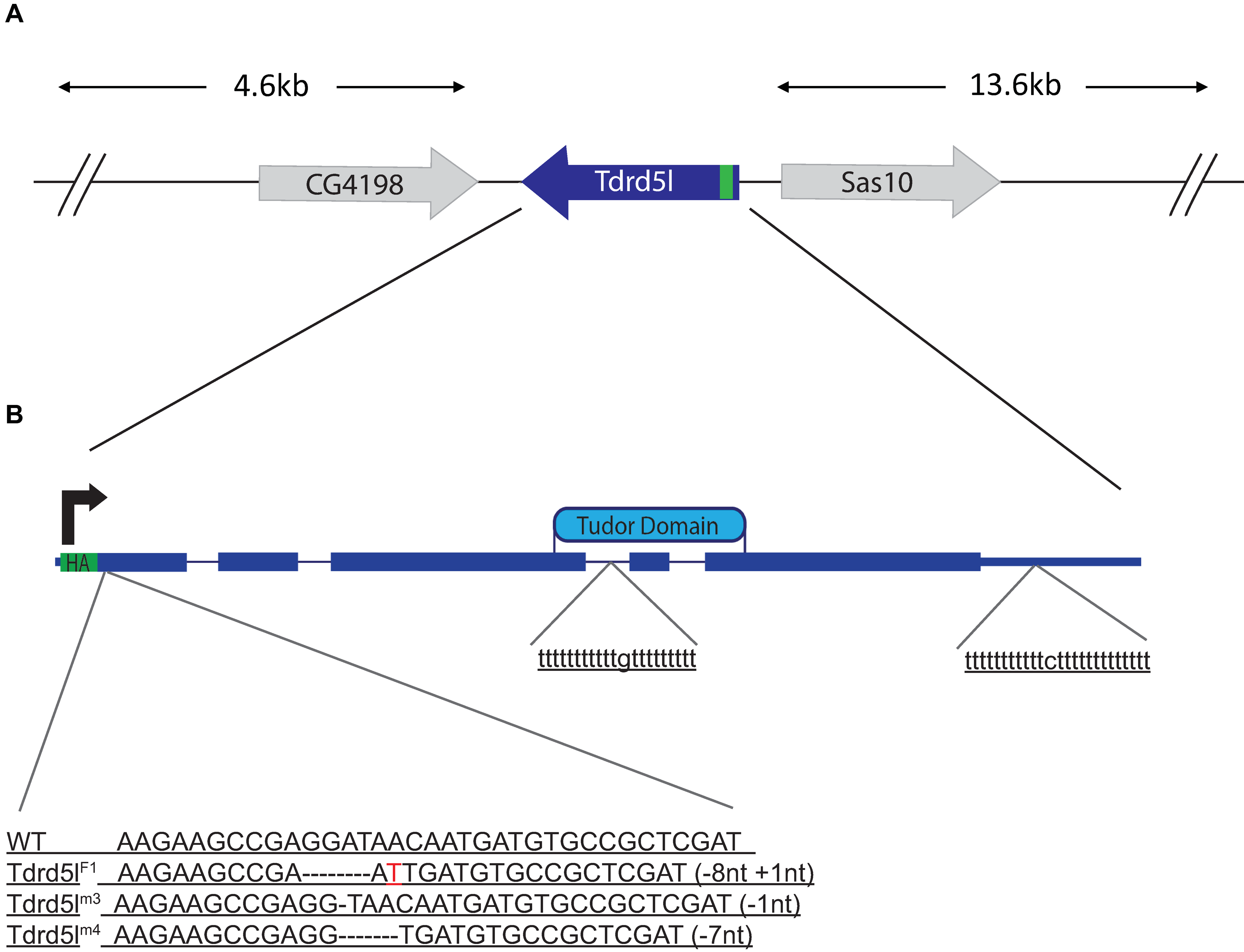

Supplement: S1 Fig — A) Schematic of the Bacterial Artificial Chromosome used to insert a hemagglutinin (HA) tag in Tdrd5l by BAC recombineering (not drawn to scale). The Tdrd5l gene is shown in reference to its closest neighboring genes: sas10 at its 5’ end and CG4198 at its 3’ end, which are both on the opposite strand. B) Gene model of Tdrd5l. The HA tag was inserted at the N-terminus of Tdrd5l immediately after the start codon. Putative Sxl binding sites are located in the 3rd intron and the 3’UTR. Mutant alleles described were generated by CRISPR-Cas9-mediated gene editing in the 1st exon. (TIF) [file pgen.1007617.s004.tif]

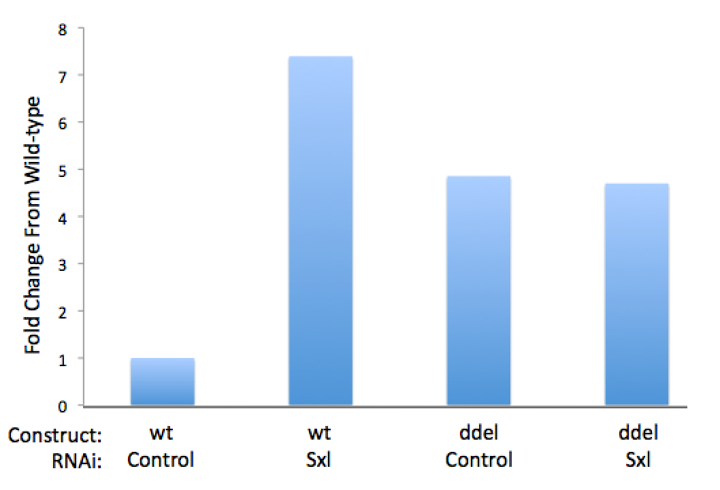

Supplement: S2 Fig — Quantification of the band intensities in Fig 3E, which confirms the repression of HA::Tdrd5l expression by Sxl. Quantification done using imageJ. Background was subtracted from each pixel intensity, then HA intensity was divided by the H3 loading control intensity. The expression of wt BAC in wt background (wt control RNAi) was set to 1 to calculate the differential expression of the other genotypes. (TIF) [file pgen.1007617.s005.tif]

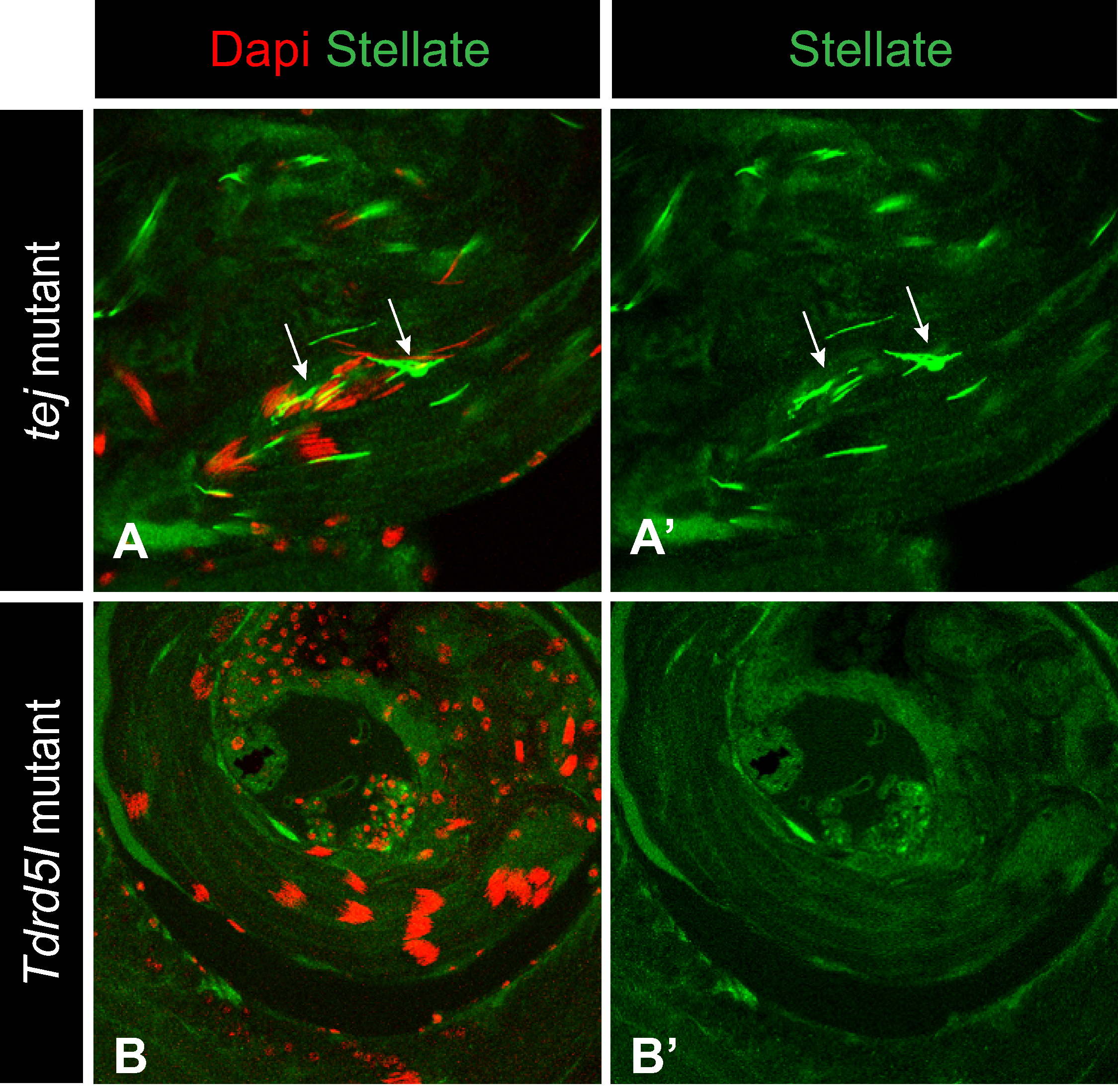

Supplement: S3 Fig — Confocal images showing expression of stellate crystals (arrows) in A) tej mutant testes but not in B) Tdrd5l mutant testes. Antibodies used are as indicated in the figure. (TIF) [file pgen.1007617.s006.tif]
